# Supplementary material for: Pan-Genomic Regulation of Gene Expression in Normal and Pathological Human Placentas
Source: Cells. 2023 Feb 10;12(4):578. doi: 10.3390/cells12040578 (PMC9954093; doi:10.3390/cells12040578)
Supplement: Supplementary file 1 [file cells-12-00578-s001.zip › cells-2044603-Figure S1.pdf]

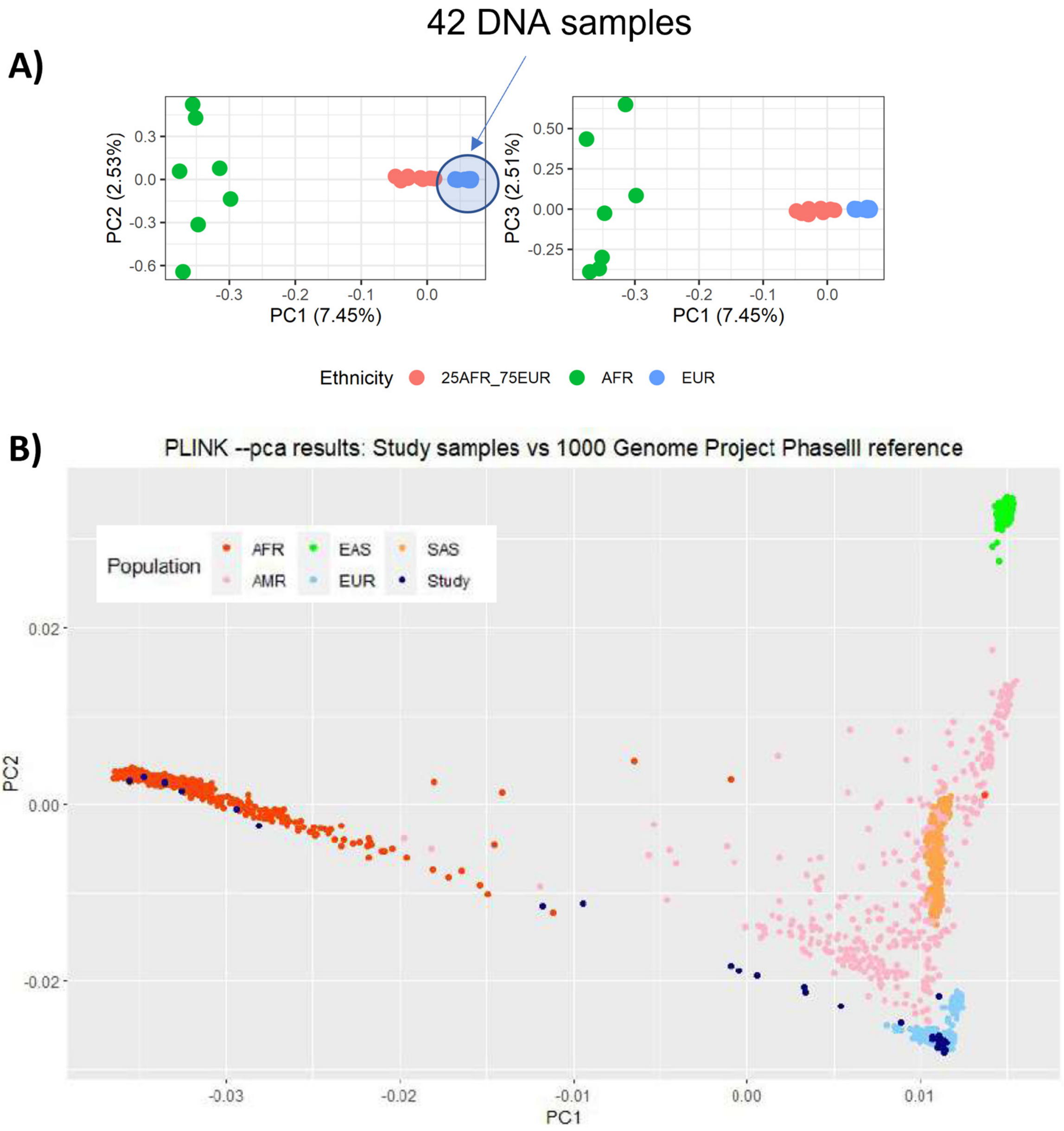

Supplementary Figure S1. Principal component analysis for population stratification and ancestry estimation. **A)** PCA plots relative to the first three PCs summarising the population stratification in the human placental samples included in the eQTL analyses. **B)** PCA plot showing PC2 vs PC1, clustering the samples of known ancestry of the 1K Genomes Project Phase III based on the differences in their allele frequencies across the tested variants, included in the pruned dataset. Placental samples are labelled in blue as "Study"; their ancestry was estimated based on the relative overlap with the groups defined by the 1K Genomes Project Phase III samples.

AMR = American; AFR = African; EUR = European; EAS = East Asian; SAS = South Asian.
